# Black Urine

**Published:** 1904-03-26

**Authors:** 


### BLACK URINE.

Dr. Garrod[1] reminds us that one of the earliest recorded facts of clinical medicine is that urine is sometimes black. Both Hippocrates and Galen mention it as a symptom of fatal significance. Doubtless many of the cases mentioned in ancient books are instances of hæmaturia or hæmoglobinuria, or of urine rendered dark by bile pigment. He quotes from the pages of Zacutus Lusitanus (1649) the interesting case of a boy who had always passed black urine. In spite of the drastic measures adopted by his medical advisers to subdue the "fiery heat" of his internal organs, to which the phenomenon was attributed, the patient lived on for many years in good health, but still passing urine as black as ink. This case was probably an example of alkaptonuria, although there are some other rare conditions in which dark urine may be passed for many years or even throughout life.

Excluding cases of jaundice, hæmaturia, and hæmoglobinuria, urine which is black when excreted, or which becomes black subsequently, may be met with in hæmatoporphyrinuria, melanotic sarcoma, alkaptonuria, ochronosis, after the taking of certain drugs (*e.g.*, carbolic acid) or articles of diet, and in cases in which abundance of indican is present, in some cases of phthisis (only after the urine has been standing for some time), and in some other rare, undeterminated conditions.

In the condition known as hæmatoporphyrinuria the urine may be black, but the dark colour is not due so much to the presence of hæmatoporphyrin as to other dark pigments which yield no characteristic spectra, and the nature of which is unknown. Most of the cases of hæmatorporphyrinuria recorded of recent years have been due to the taking of sulphonal or its allies (trional) over long periods, but occasionally the condition occurs apart from any drug administration, and one example has been reported of paroxysmal hæmatoporphyrinuria with symptoms like those of paroxysmal hæmoglobinuria. The recognition of such urines depends upon the observation of the spectroscopic absorption bands; as there is usually no albumen in the urine the presence of hæmoglobin can readily be excluded. Urine, whatever its reaction, never shows the spectrum of acid hæmatoporphyrin unless a mineral acid has been added to it.

True melanuria, associated with melanotic sarcoma, is rare. Dr. Garrod's observations are confirmatory of the following points:—In cases in which the growth is limited to its primary seat, or has not reached beyond the neighbouring lymphatic glands, melanogen (the chromogen of melanin) is not found in the urine. Only when the internal viscera have become involved does melanuria result, and the degree of melanuria appears to be proportional to the extent to which the liver is involved, and to the quantity of pigment in the growths in that organ. He has never found melanuria in the absence of growths. Usually the urine is of normal colour when passed; but on standing exposed to the air it quickly becomes brown and then black, darkening taking place first at the surface and then spreading downwards. The addition of nitric acid to the cold urine at once produces blackness. Ferric chloride also causes immediate blackening, and a grey precipitate which forms is redissolved by excess of the reagent. This is the most delicate and distinctive test for the presence of melanogen.

In alkaptonuria, as in melanuria, the urine is normal in colour when fresh, but darkens, from the surface downward, on standing. The change occurs much more rapidly when an alkali is added, a reaction which has given the condition its name. Alkaptonuria is an extremely rare anomaly of proteid metabolism, usually congenital and lifelong, and one which appears to be harmless. Of the recorded cases a number have occurred in brothers and sisters; only two instances of direct transmission from parent to child are known, and a large proportion of the known alkaptonurics have been the offspring of consanguineous marriages. The condition appears to depend upon an incomplete destruction of certain products of proteid metabolism, namely, tyrosin and phenylalanin, which therefore, in an altered form (homogentisic acid), appear in the urine. The condition is recognised by the characters already described, and by the fact that the urine reduces Fehling's solution with the aid of heat, and ammoniacal silver nitrate solution in the cold. Hence the patient may be thought to suffer from diabetes.

Ochronosis is an extrememly rare condition, no case having as yet been recorded in this country. It apparently has some affinities with alkaptonuria. Pigmentation occurs during life in the cartilages and some other tissues (*e.g.* conjunctiva).

Indicanuria, although among the commoner causes of dark urine, is, Dr. Garrod believes, not nearly so well recognised as it should be. In this condition the urine blackens when warmed with nitric acid, but there is no such immediate blackening in the cold as occurs in true melanuria. No blackening is caused by the addition of ferric chloride, by which test the presence of melanogen is excluded. A confirmatory test is afforded by heating the urine with hydrochloric acid and a drop of nitric acid, and shaking with chloroform, by which means the indigo pigments are readily extracted, although the supernatant liquid retains an inky blackness. Such urines do not reduce Fehling's solution. Conditions which lead to abundant secretion of indican are intestinal obstruction, excessive bacterial activity in the intestine, putrefactive changes in collections of pus.

It was observed by Hale White that in some cases of phthisis the urine, even if kept for weeks exposed to the air, remained acid, and sometimes became brown or black on long keeping. This phenomenon may be due to the increased excretion of phenols, which has been shown to take place in cases of advanced phthisis in which decomposition changes are going on in the cavities.

Drugs which may cause darkening of the urine are carbolic acid, naphthalin, salol, creasote, thallin, and in a less degree the salicylates. Carboluria is produced so readily in children that it often follows the application of a carbolic compress to the scalp for the destruction of pediculi. The dark colour of the urine in this case is due to hydroquinone.

